# Quality of life of children with residual seizures after epileptic resection surgery

**DOI:** 10.3389/fneur.2022.1066953

**Published:** 2022-12-21

**Authors:** Yuxin Wu, Zaiyu Zhang, Ping Liang, Bin Zou, Difei Wang, Xuan Zhai

**Affiliations:** ^1^Department of Neurosurgery, Children's Hospital of Chongqing Medical University, National Clinical Research Center for Child Health and Disorders, Ministry of Education Key Laboratory of Child Development and Disorders, Chongqing, China; ^2^Chongqing Key Laboratory of Translational Medical Research in Cognitive Development and Learning and Memory Disorders, Chongqing, China

**Keywords:** epilepsy, epilepsy surgery, quality of life, family, children

## Abstract

**Objective:**

Epilepsy dramatically affects the quality of life (QoL) of children, and resection surgery can improve their QoL by reducing seizures or completely controlling them. Children who have postoperative seizures tend to show a poorer QoL. The aim of the present study was to investigate the QoL of children with seizures after resection surgery and its influencing factors.

**Methods:**

In the present study, we retrospectively reviewed 151 consecutive children who underwent resection surgery. We then divided them into two groups, seizure and seizure-free groups, according to the seizure outcomes 1 year after surgery. Variables were categorized into a number of factor types such as preoperative factors, surgery-related factors, postoperative factors, and family factors. QoL and seizure outcomes more than 3 years after surgery were assessed according to the ILAE seizure outcome classification and the CHEQOL-25 scale.

**Results:**

Forty-three (28.5%) of the 151 children had seizures 1 year after surgery, and two children died during the follow-up period. The mean CHEQOL-25 scale for children with seizures was 63.5 ± 18.2, and 20 (48.8%) patients had poor QoL. Surgery-related factors, such as surgical complications and surgical sequelae, were not statistically associated with QoL. Preoperative language development retardation or language dysfunction [odds ratio (OR) = 29.3, *P* = 0.012) and postoperative ILAE seizure outcome classification (OR = 1.9, *P* = 0.045)] were significantly associated with QoL.

**Significance:**

Children with seizures after resection surgery had a relatively poor QoL. Surgery-related factors, such as surgical complications and surgical sequelae, cannot predict the QoL. Preoperative language development retardation or language dysfunction and postoperative ILAE seizure outcome classification were independent predictors of the quality of life (QoL). For children who could not achieve the expected freedom from seizure after surgery, a lower ILAE grade (ILAE 1-3) is also an acceptable outcome since it predicts a higher QoL.

## Keypoints

- Nearly half of children with epilepsy who have postoperative seizures display a poor quality of life (QoL).
- Presurgical language development delay or language dysfunction and the postsurgical International League Against Epilepsy (ILAE) seizure outcome classification can predict the QoL for children with epilepsy who have postoperative seizures.
- Other varied factors such as the annual household income and surgery-related factors, such as surgical complications, surgical sequelae and surgical type, are not associated with QoL and so do not make any significant difference in QoL for children with postoperative seizures.
- If seizure freedom is difficult to achieve after surgery, then a lower ILAE seizure outcome classification, such as ILAE 1-3, is still an acceptable outcome, as it predicts a higher QoL.

## 1 Introduction

Epilepsy is a common chronic neurological disorder that affects cognition, memory, behavior, emotion, and the quality of life (QoL) of pediatric patients (1, 2). Due to the sudden, recurrent and unpredictable nature of seizures, the effects of epilepsy in children extend beyond the direct effects of seizures themselves (3). Seizures affect QoL in a number of ways, including 1. Physical impairments: accidental falls during seizures, weakness postseizure, and dizziness reduce the patient's self-caring ability (4); 2. Cognitive impairments: seizures, anti-epileptic drug (AED) side effects, and interictal discharges have a negative impact on the patient's cognitive function and memory (5–7); 3. Psychological impairments: feelings of shame, social impairment, and social isolation associated with seizures have a considerable psychological and emotional impact (8, 9); and 4. Social function impairments: most children with epilepsy have a low educational status, while people with epilepsy also have much higher unemployment rates than the general population (10). These factors mainly account for the reduced QoL of people with epilepsy. It is important to clarify that the ultimate goal of epilepsy surgery is to improve QoL rather than just to control seizures.

Previous studies showed that, postoperatively, the QoL of children with epilepsy improved compared to presurgery (11, 12), and postoperative seizure outcomes, especially freedom from seizure, are the best predictors of QoL in children. A meta-analysis suggested that children achieving freedom from seizure after surgery showed significant postoperative QoL improvement, while children with residual seizures did not improve substantially postoperatively (13). Thus, seizure freedom was regarded as the central desirable outcome postoperatively, as it predicts a meaningful improvement in QoL. Nevertheless, some studies also reported an improvement in QoL in a proportion of patients with residual seizures after surgery (14). Considering that nearly one-third of children with epilepsy cannot achieve freedom from seizure after resection surgery (15), it is meaningful to explore the long-term QoL and its influencing factors in such a large group, which can provide prognostic information and improve postoperative management. Therefore, the present study aimed to investigate the QoL of children with seizures 1 year after resection surgery and explore independent predictors of postsurgical QoL to guide clinicians in treatment planning.

## 2 Methods

Children (< 18 years) with epilepsy who underwent resection surgery at the Department of Neurosurgery, Children's Hospital of Chongqing Medical University between July 2015 and July 2019 were included in this retrospective study. We measured the seizure outcomes of all children 1 year after surgery and their family characteristics, as well as current seizure outcomes and QoL of children with postoperative seizures. The exclusion criteria were as follows: 1. Children who underwent a palliative surgery (neurostimulation, corpus callosotomy, and so on) and hemispherectomy; 2. Children who had undergone surgery for epilepsy previously; and 3. Children with epilepsy syndrome or epileptic encephalopathy. Because children with epilepsy usually have the expected hemiparesis after hemispherectomy (15). which has a significant impact on QoL, and previous studies have proven that neurostimulation provides positive effects on QoL and emotional and behavioral outcomes (14), our cohort excluded patients undergoing palliative surgery and hemispherectomy. Epilepsy syndrome or epileptic encephalopathy is usually related to a severe neurodevelopmental delay. The neurodevelopmental delay caused by the above two diseases themselves has a great impact on the QoL. Thus, our analysis cohort excluded children with epileptic syndrome and epileptic encephalopathy. Our surgical procedure has been described in a previous study about a nomogram prediction model for predicting postsurgical seizure outcomes in children with focal cortical dysplasia (FCD). We localize the epileptic focus based on a combination of symptoms, physical examination, magnetic resonance imaging (MRI), and video electroencephalogram (EEG) results prior to surgery. In cases where the epileptic focus cannot be precisely located or is suspected to involve an eloquent area, fluorine-18-labeled-fluorodeoxyglucose-PET (^18^F-FDG-positron emission tomography) and invasive EEG were performed to identify the epileptic focus. After an elaborate neurological examination, delayed language development and delayed motor development were diagnosed based on the Gesell Developmental Scale. We extracted hospitalization and follow-up medical records and evaluated the seizure outcomes at 1 year postoperatively in children who underwent resection surgery. Last, we assessed the current seizure outcomes and quality of life over the follow-up period of at least 3 years in patients who still had seizures 1 year after surgery.

Ethical approval was given by the Medical Ethics Committee of Children's Hospital of Chongqing Medical University, and informed consent was obtained from the caregivers of children.

### 2.1 Data collection

Demographic data, seizure-related information (duration, seizure type, and seizure frequency), the dose and regimen of anti-epileptic drugs (AEDs), and surgery-related information were collected in this study. The latest frequency of seizures was classified into daily, weekly, monthly, or less, and seizure types were classified as generalized tonic–clonic seizures (GTCS) and others. Unanticipated adverse events related to the surgery such as infection, intracranial hemorrhage, and hydrocephalus were considered surgical complications, and as opposed to this, some expected postoperative adverse events were considered part of the common surgical outcome rather than a complication, such as hemiplegia after hemispherectomy. New-onset, unanticipated neurological deficits after surgery were regarded as sequelae that were classified as long-term or short-term sequelae according to whether they recovered within 6 months after surgery.

The family characteristics we investigated included the education level of the caregiver and the income of the family. We collected information on the annual income of the family and the caregiver's education level with the caregiver's consent to model the influence of disposable medical resources and the caregiver's cognition of epilepsy on children's QoL.

### 2.2 Outcome measures

The seizure outcomes of children with epilepsy were evaluated by the International League Against Epilepsy (ILAE) seizure outcome classification 1 year after surgery (16). The QoL of children with epilepsy after surgery was assessed using the CHEQOL-25 scale, a QoL assessment tool proposed by Gabriel Ronen, which evaluated five aspects of epilepsy: “Present Concerns,” “Interpersonal Emotion,” “Secrecy,” “Normality,” and “Future Worries.” Subjects responded to five questions for each aspect and a total percentage score for each item was calculated, with higher scores representing a better QoL (17). The CHEQOL-25 scale is being used in children with epilepsy postoperatively in some developing countries in Southeast Asia (18). The questionnaire is designed to be filled out either by the children or by the caregivers, but, as the children in the present study had seizures, we elected to have the questionnaire filled out by the caregivers. Referring to the recommendations of the inventors of the CHEQOL-25 scale, we categorized the QoL into two grades: average or better (total scores 60+) and poor (total scores < 60).

### 2.3 Data analysis

Data were analyzed using the SPSS 27.0 software. Continuous variables were summarized using the means and standard deviations (SDs) and categorical variables were expressed in numbers (%). To study the effect of disposable medical resources on the patient's QoL, we referred to the data from the China Statistical Yearbook, which show an annual per capita income of 35,000 ¥ in China. After collecting details on the annual income of caregivers, we grouped them into two types, caregivers with more than average income and caregivers with less than average income, according to the annual household incomes >70,000 and < 70,000 (We considered two household members participating in work per family). The education level of caregivers was categorized according to whether they had a bachelor's degree or higher. The univariate analyses of categorical variables were conducted *via* Pearson's chi-square test and Fisher's exact test. The univariate analyses of continuous variables were conducted using the non-parametric Mann–Whitney U-tests to screen for variables that might affect QoL. *P-*values were adjusted using the Benjamini and Hochberg procedure along with variables. A *P* ≤ 0.10 was included in the multivariate logistic regression model and odds ratio (OR) values and 95% confidence intervals (CIs) were recorded, with a *P* < 0.05 considered significant.

## 3 Outcomes

### 3.1 Demographic data

**Figure 1:**
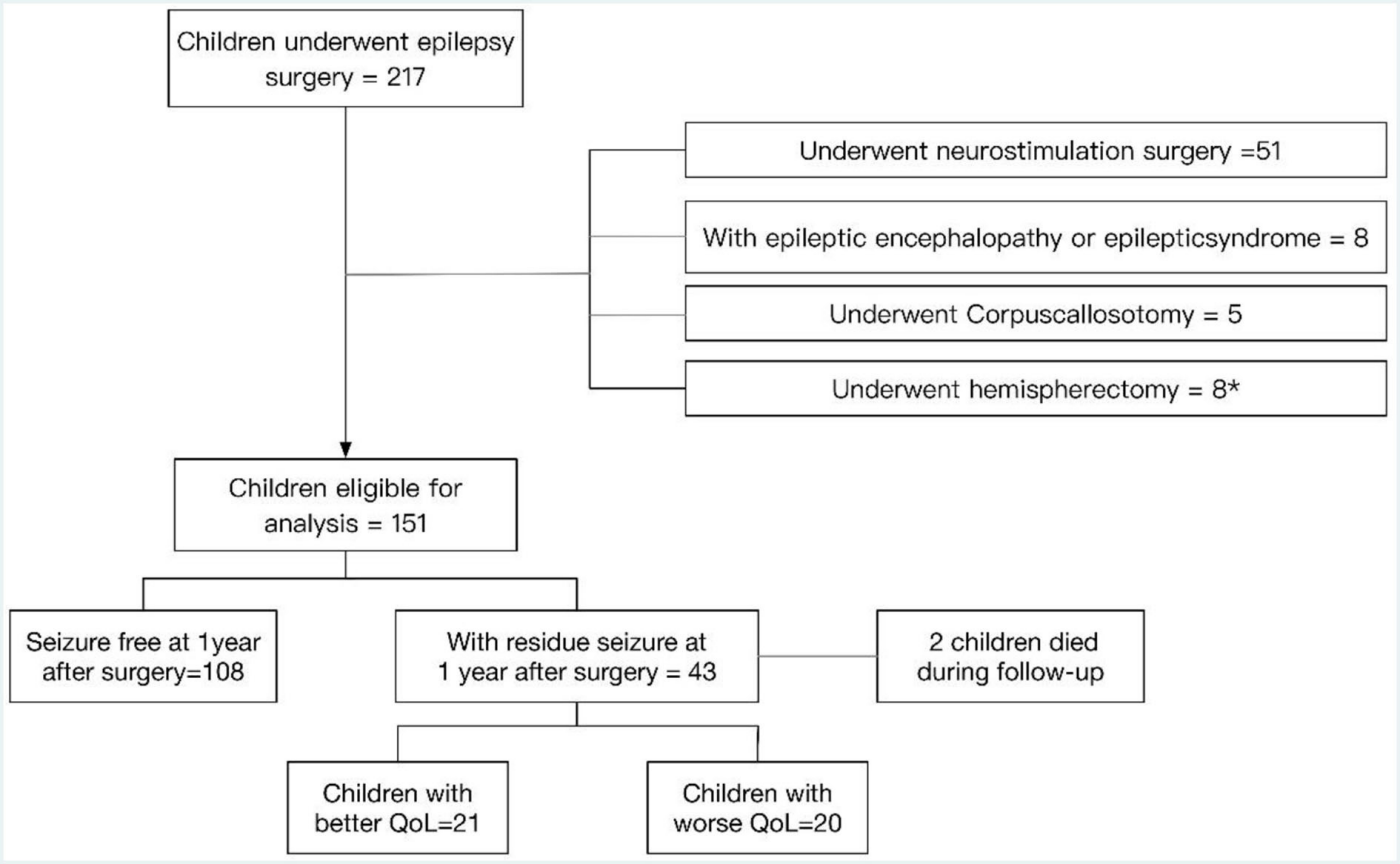
Patient selection flowchart. *Six of the eight children who underwent hemispherectomy were diagnosed with epilepsy syndrome.

A total of 217 children underwent epilepsy surgery, out of which 51 children underwent neurostimulation surgery, eight children had epileptic encephalopathy or epileptic syndrome, five children underwent corpus callosotomy and eight children underwent hemispherectomy. Out of the latter eight children, six of them who underwent hemispherectomy were diagnosed with epilepsy syndrome. A total of 151 children with epilepsy who were eligible for analysis and who underwent resection surgery at the Children's Hospital of Chongqing Medical University from July 2015 to July 2019 were enrolled in the present study. They were followed for at least 3 years after surgery and subsequently followed with a median follow-up of 5 years. The process of inclusion in the current study is presented in the flow diagram, a patient selection flowchart, in Figure 1. One hundred eight children remained seizure free for 1 year after surgery. Forty-three (28.5%) children had seizures at 1 year after surgery, two children with postoperative seizures died during follow-up, and details of the 41 survivors were recorded in an additional file. There were 21 children with better QoL and 20 children with worse QoL. The proportion of males and females was approximately equal, with 56.1% male and 43.9% female. The median age at the onset of epilepsy was 6 years and the median age at surgery was 8 years. Out of 41 patients with postoperative seizures, 8 were admitted (19.5%) for pathology type focal cortical dysplasia type II (FCDII), 7 (17.1%) for pathology type focal cortical dysplasia type I (FCDI), 6 (14.6%) for encephalomalacia, 15 (36.6%) for brain tumors, 4 for (9.8%) for vascular malformations, and 1 for (2.4%) for gray matter heterotopias (Table 1). All 41 children were born in two-parent families, of which 63.4% (*n* = 26) of families had an annual household income greater than the average annual income in China and 26.8% (*n* = 11) of caregivers had obtained a bachelor's degree or higher.

**Table 1:** Characteristics of children with residual seizure after epileptic resection surgery.

**Characteristic**	**Number of patients (%)/** **Mean ±SD (range)**
Patients	41
Male	23 (56.1%)
Age at onset (year)	5.90 ± 3.97 (0–9.0)
Age at surgery (year)	7.41 ± 3.90 (1.0–13.0)
Duration of epilepsy (year)	1.54 ± 2.46 (0–9.0)
**Cause of epilepsy**	
Tumor	15 (36.6%)
FCD	15 (36.6%)
Vascular malformations	4 (9.8%)
Other^a^	7 (17.1%)
**Seizure frequency, 1 year after surgery**	
Daily	18 (43.9%)
Weekly	10 (24.4%)
Monthly or less	13 (31.7%)
**Seizure frequency, latest**	
Daily	27 (65.9)
Weekly	7 (17.1%)
Monthly or less	7 (17.1%)

SD, standard deviation; FCD, focal cortical dysplasia.

^a^Other: Gray matter heterotopias: one patient; encephalomalacia: six patients.

### 3.2 Seizure outcomes

**Figure 2:**
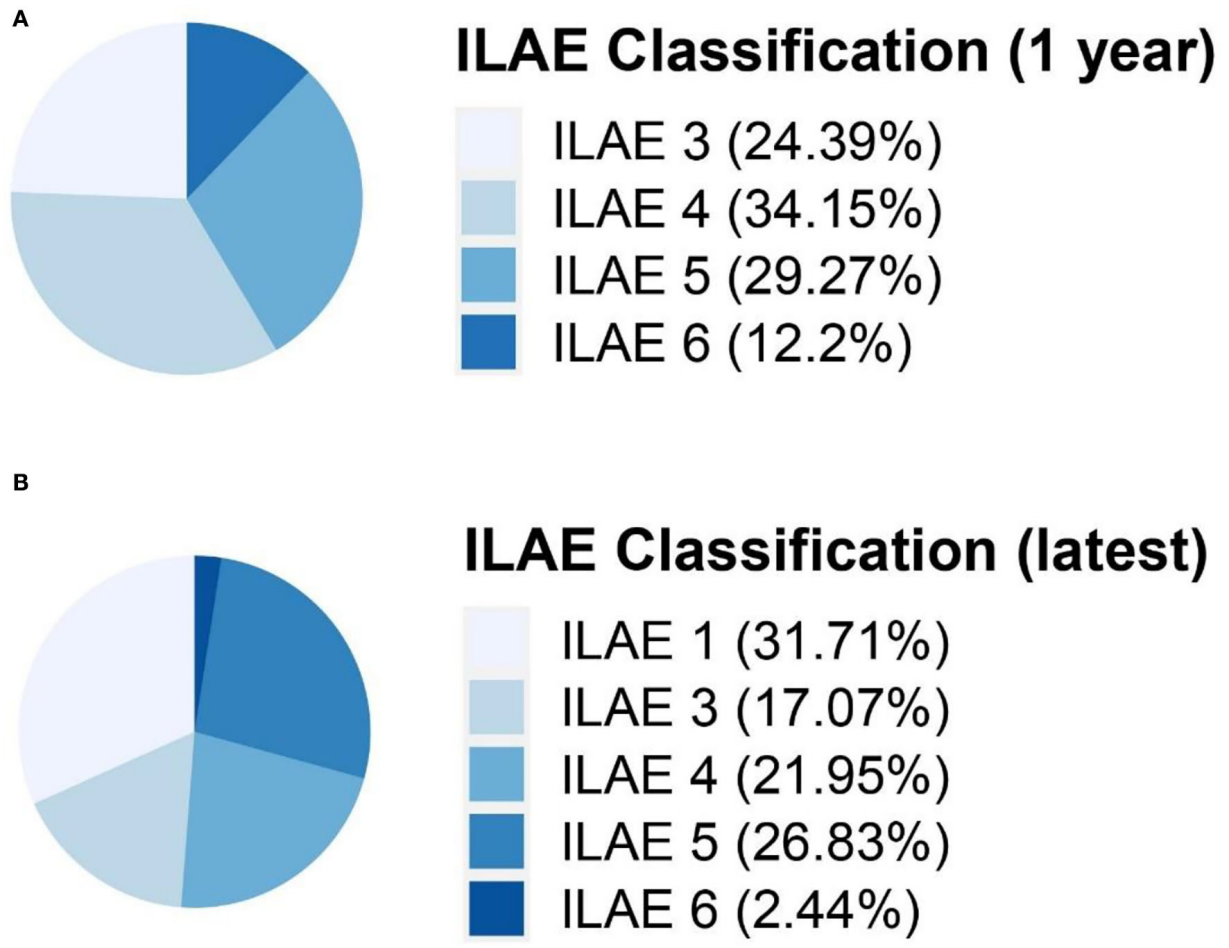
Seizure outcomes according to ILAE classification 1 year after surgery **(A)** and latest **(B)**. ILAE classification, International League Against Epilepsy seizure outcome classification.

All 41 participants had seizures 1 year after surgery, 58.5% (*n* = 24) with ILAE 3-4 and 41.5% (*n* = 17) with ILAE 5-6. The seizure outcomes according to ILAE classification 1 year after surgery and latest are presented in Figure 2. After a median follow-up of 5 years, 31.7% (*n* = 13) with ILAE 1 achieved freedom from seizure, 58.5% (*n* = 24) with ILAE 4-5 had better seizure outcomes than 1 year postoperatively, 9.8% (*n* = 4) had more frequent seizures than 1 year postoperatively, and 31.7% (*n* = 13) with ILAE 1 had no significant change in seizures from 1 year after surgery, as shown in Figure 2. The absence of complete resection of the epileptogenic focus (*P* < 0.01) was statistically associated with seizure freedom at follow-up. 48.0% of children with complete resection achieved seizure freedom at follow-up, while only 6.3% of patients with incomplete resection achieved seizure freedom, and the distributions in the two groups differed significantly (chi-squared = 7.9, in the complete resection group, seizure free: residual seizure = 12:13, *P* = 0.005 two-tailed).

### 3.3 Quality of life at follow-up

**Figure 3:**
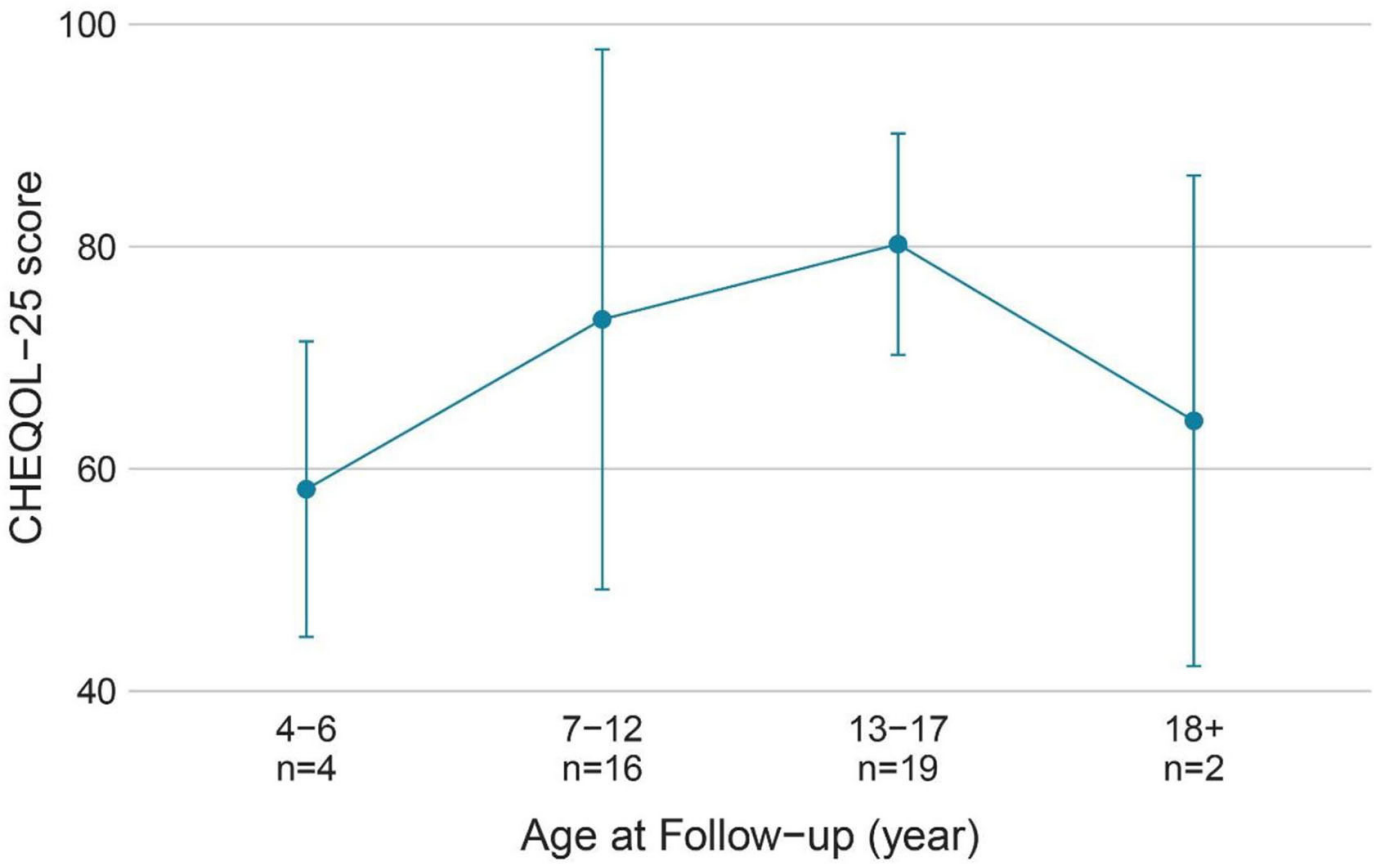
QoL (CHEQOL-25 total score) group means (±standard error) of patients of different age groups. QOL, quality of life.

**Figure 4:**
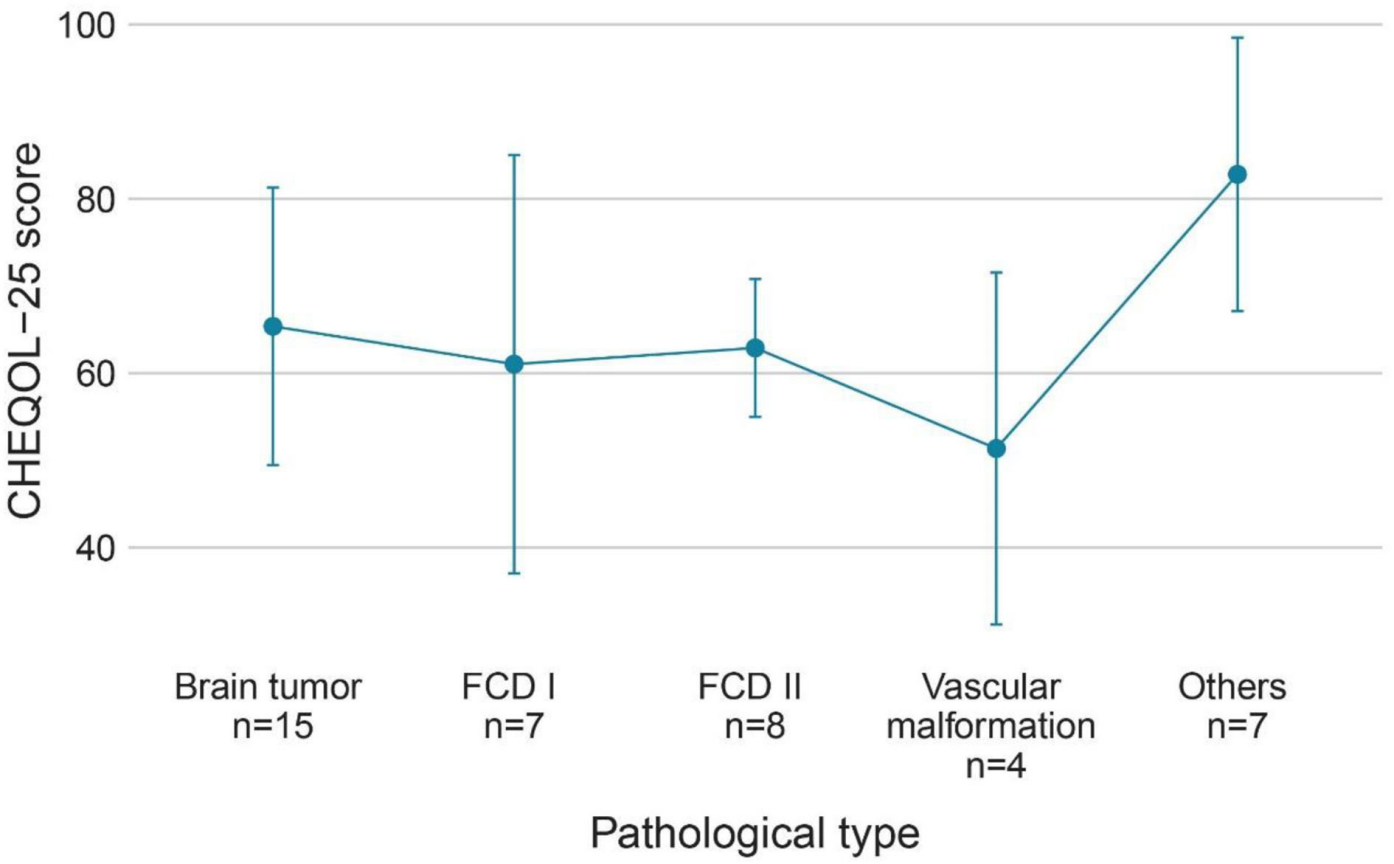
QoL (CHEQOL-25 total score) group means (±standard error) of patients with different pathologic types. QOL, quality of life.

**Figure 5:**
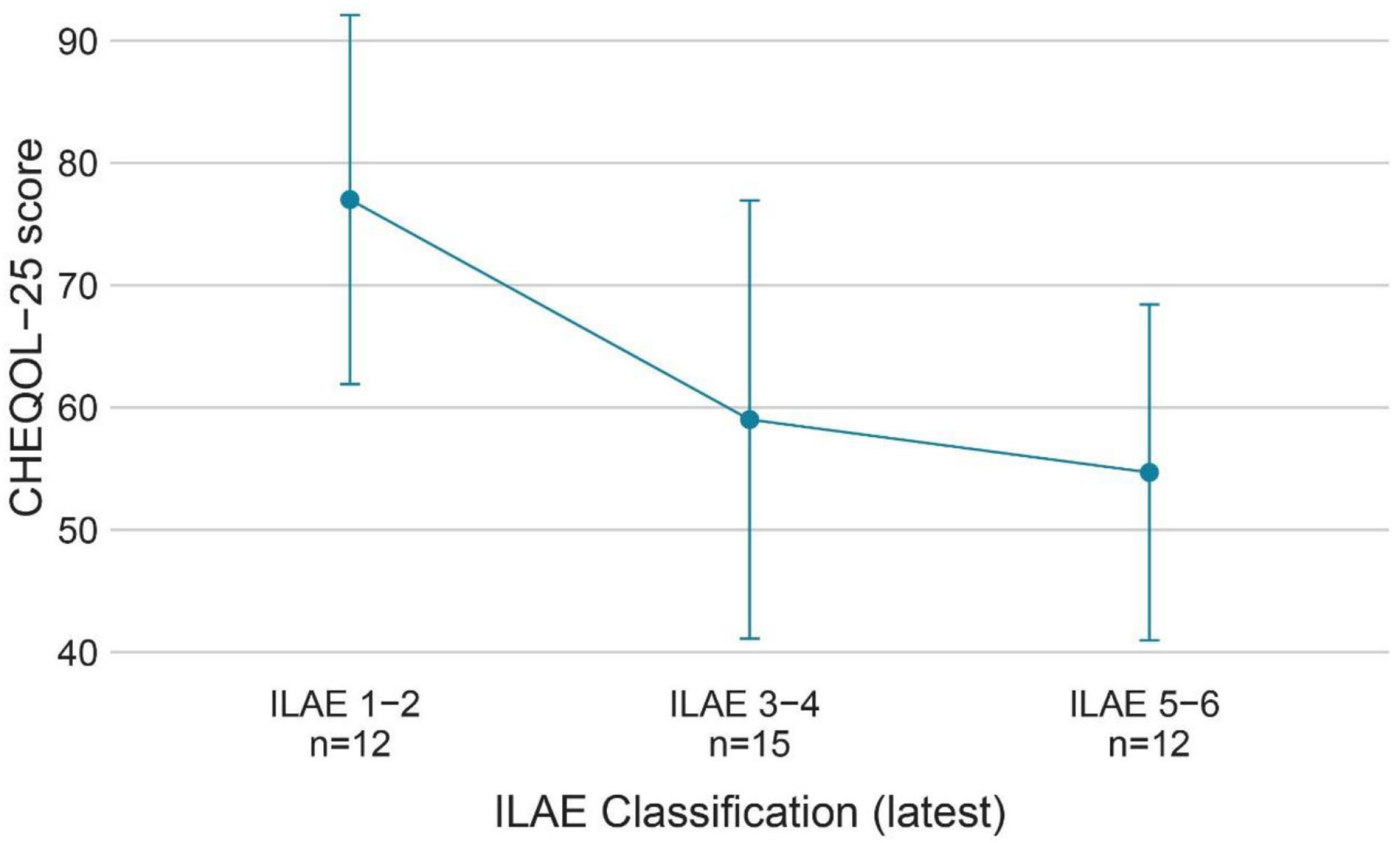
QoL (CHEQOL-25 total score) group means (±standard error) of patients with different ILAE seizure outcome classifications (latest). QOL, quality of life; ILAE classification, International League Against Epilepsy seizure outcome classification.

The QoL score for all ages was 63.5 ± 18.2 (range: 13.9–92.2), and the number of children with a poorer quality of life (*n* = 20) was similar to that with better QoL (*n* = 21). Figure 3 shows the QoL of patients in different age groups, and Figure 4 shows the QoL (of patients in different pathologic types. Figure 5 shows the QoL of patients with different ILAE seizure outcome classifications (latest). The proportion of children with or without preoperative language developmental delay having poor QoL after epilepsy surgery was 90 and 35.5%, respectively, and the distributions in the two groups differed significantly (chi-squared = 6.9, good QoL: poor QoL = 1:9, adjusted *P* = 0.10 two-tailed). Since the ILAE seizure outcome classification pertained to graded data, the Mann–Whitney U test was used for analysis. The results showed that there was a significant difference in ILAE seizure outcome classification 1 year after surgery (Mann–Whitney U = 122.5, good QoL: poor QoL= 21:20, adjusted *P* = 0.10 two-tailed) and the latest ILAE seizure outcome classification (Mann–Whitney U = 122.0, good QoL: poor QoL= 21:20, adjusted *P* = 0.10 two-tailed) between the good quality of life group and the poor quality of life group (Table 2). These three variables were included in a multivariate logistic regression model, and presurgical language development delay or speech dysfunction (OR = 29.3, *P* = 0.012) and ILAE seizure outcome classification at follow-up (OR = 1.9 *P* = 0.045) were independent predictors of QoL for children with postsurgical seizures (see Table 3).

**Table 2:** Variables associated with a meaningful, clinically important change of QoL in children with residual seizure after resection surgery.

**Variable**	**Better** ** QOL** ** (*n* = 21)**	**Worse** ** QOL** ** (*n* = 20)**	**Statistics** ** (Chi-square** ** or z-value)**	**P-value**	**Adjusted** ** P-value**
**Demographics**					
Language development retardation	1 (10.0%)	9 (90.0%)	6.94	0.008	0.10*
Motor development retardation	3 (37.5%)	5 (62.5%)	0.22	0.64	0.84
Age at surgery (year)	8.0 (1.5–10.0)	8.0 (5.3–11.0)	−0.84	0.40	0.67
Duration of epilepsy (year)	1.0 (0–1.5)	0.6 (0–3.0)	−0.39	0.70	0.84
**Epilepsy before surgery**					
Polypharmacy	6 (60.0%)	4 (40.0%)	0.08	0.78	0.84
Seizure frequency			−0.45	0.65	0.84
Epilepsy-related injury	0 (0.00%)	0 (0.00)	-	-	-
**Surgery-related factors**					
Multilobar resections	5 (31.3%)	11 (68.8%)	4.19	0.04	0.15
Surgery complication	9 (56.3%)	7 (43.8%)	0.08	0.78	0.84
Long term sequelae	2 (22.2%)	7 (77.8%)	2.54	0.11	0.28
**Epilepsy after surgery**					
Seizure frequency, latest			−1.18	0.24	0.51
Polypharmacy	11 (52.4%)	10 (47.6%)	0.08	0.88	0.88
ILAE, 1 year after surgery	-	-	−2.38	0.02	0.10*
ILAE, latest	-	-	−2.38	0.02	0.10*
**Family factor**					
Low family income	5 (33.3%)	10 (66.7%)	3.03	0.08	0.24
Caregiver with bachelor's degree or higher	7 (63.6%)	4 (36.4%)	0.93	0.34	0.64

QoL, quality of life; ILAE classification, International League Against Epilepsy seizure outcome classification; EEG, electroencephalogram; GTCS, Generalized tonic–clonic seizures.

*Indicates significant differences.

**Table 3:** Multivariate logistic regression models showing the variables independently associated with the meaningful changes in QoL in children with residual seizure after resection surgery.

**Variable**	**OR**	**OR 95%CI**	**P-value**
Language retardation	29.3	2.10–409.06	0.012*
ILAE_1year	1.25	0.49–3.15	0.641
ILAE_latest	1.92	1.02–3.65	0.045*

QOL, quality of life; ILAE classification, International League Against Epilepsy seizure outcome classification.

*Indicates significant differences.

## 4 Discussion

Quality of life is defined as “individuals' perception of their position in life in the context of culture and value systems in which they live and in relation to their goals, expectations, standards, and concerns” (19). The assessment of QoL focuses on the individual's subjective perception of wellbeing, which is more representative of the patient's role functioning, overall health, and satisfaction with medical care than seizure outcomes (20). Epilepsy affects the quality of life in emotional, physical, social, and spiritual dimensions (21). The goals of epilepsy treatment have shifted such that the QoL in children with epilepsy is now routinely assessed in many medical centers (22).

The present study found that children with seizures 1 year after surgery (ILAE grade 2-6) had a mean QoL score of 63.5 ± 18.2 (range: 13.9–92.2) after a median follow-up time of 5 years. Nearly half of the patients had poor QoL. These results corroborate the findings of Wu et al., who reported a mean QoL score of 65.6 ± 14.1 in Chinese adolescents with epilepsy in a previous study, but their study included patients with epilepsy who received medication treatment in 2005 (23). Overall, the QoL of children with postoperative seizures was consistent with previous data but it seems to be still unsatisfactory.

A prospective study of children who underwent temporal lobe epilepsy surgery showed that seizure freedom was a good predictor of QoL, whereas preoperative factors could not predict QoL (24). In accordance with the results, our study found that preoperative factors, such as preoperative seizure type, seizure frequency, and anti-epileptic drugs (AEDs), were not statistically associated with QoL. Surgery-related factors such as surgical complications, incomplete resection, and surgical sequelae, as well as household income and caregiver education level, were not statistically associated with QoL. This is a somewhat disappointing finding which suggests that once a child has postoperative seizures, we cannot then predict QoL *via* complications, sequelae, seizure type, seizure frequency, or other factors; thus, it is more difficult to improve the QoL. In the present study, QoL decreased as the ILAE seizure outcome classification increased, and the ILAE seizure outcome classification was an independent predictor of QoL. Increased seizures have negative effects on neurodevelopment and cognition in children, and increased seizures also make it more difficult for children with epilepsy to participate in social activities, to complete education in school, and to escape epilepsy-associated stigma. Uncontrolled epilepsy is usually accompanied by a heavier anti-epileptic drug (AED) burden and greater cognitive impairment from anti-epileptic drugs (AEDs). From such a perspective, a higher ILAE seizure outcome classification contributes to a reduced QoL (7, 25–27). There is increasing recognition of the fact that improving QoL in epilepsy is the paramount goal for patients and healthcare providers (28). Previous studies only focused on the entire epilepsy surgery cohort and found a significant improvement in the QoL of patients who achieved seizure freedom, while no improvements were noticed in patients with postoperative seizures (13). Previous studies proposed that seizure freedom is the most desirable outcome for QoL improvement after surgery (13, 27). However, this study redefined “surgical failure” because, even in children with postoperative seizures, nearly half of them could achieve a good quality of life. Considering that approximately one-third of the patients had residual seizures after surgery (15), it would be unfair to directly classify them as surgical failures when they still have the potential to improve their quality of life. In children with postoperative seizures, we could decrease the ILAE seizure outcome classification by adjusting postoperative medication and performing neurostimulation or secondary surgery. If seizure freedom cannot be achieved after surgery, a lower ILAE seizure outcome classification, such as ILAE grade 1-3, is still acceptable, as it predicts a better QoL. In our cohort, children with ILAE seizure outcome classifications 1-3 had a mean CHEQOL-25 score of 70.9 ± 17.1, while patients with ILAE 4-6 had a mean CHEQOL-25 score of 56.4 ± 16.6. This finding provides a theoretical basis for improving postoperative QoL in children with residual seizures after surgery and completing the postoperative management of epilepsy.

In our study, presurgical language development delay or language dysfunction predicted a lower QoL in patients with postsurgical seizures, while presurgical motor development delay was not statistically associated with QoL. This may be a feature of pediatric epilepsy, where epilepsy onset in childhood has a large impact on intellectual development. Fifty-three percent of children with epilepsy onset before the age of 2 years have a combined intellectual disability of 7 years (29). A total of 24.4% of children in our cohort had delayed language development or language dysfunction. Furthermore, the improvement in speech development after epilepsy surgery was not significant. Westerveld reported that only 9% of children who underwent surgery for temporal lobe epilepsy showed improvement in language development. Similar findings have been reported in children who underwent surgery for frontal lobe epilepsy (30). This may be attributable to an abnormal neural substrate or the effects of epilepsy prior to surgery that hinder the long-term capacity for language function improvement (31). Although language skills were independent of epilepsy surgery, they were associated with seizure control (31). Considering that the children in our cohort all had residual seizures after surgery, they were less likely to obtain an improved language function, and a poorer language function predicted a poorer quality of life (7).

To date, several studies reported that people with epilepsy are at risk of a seizure-related injury when engaging in risky activities such as ironing, cooking, cycling, and driving. These seizure-related injuries can cause physical damage and significantly reduce the QoL (32, 33). Unlike adults with epilepsy, children are less likely to engage in the abovementioned risky activities, but some studies reported the risk of seizure-related injury in children too (34). Children have a high need for recreation and sport, and appropriate participation in low-risk recreational and sporting activities contributes to improving the physical and mental health of children with epilepsy, but safety is a concern for the caregivers. Our study investigated the impact of physical activity on QoL. Thirty-eight (92.7%) out of the 41 children attended school normally. Thirty-six (87.8%) children participated in physical education classes and were engaged in regular outdoor exercise, and no child reported any accidental injury from physical activity at follow-up. Hence, it could conceivably be hypothesized that the seizure-related injury has a lesser impact on the QoL in children with epilepsy. This also suggests that children with seizures can participate in low-risk physical activities under the close supervision and monitoring of caregivers.

The annual household income and caregiver's education level were chosen to represent the impact of household disposable medical resources and the caregivers' understanding of epilepsy on the QoL of children. We found no statistically significant difference between the QoL of children with epilepsy who underwent epilepsy surgery in families with lower incomes and in those with higher incomes. However, rather than completely denying the influence of economic factors on the quality of life, we considered these findings to be in line with the actual situation in China, but these findings should be carefully generalized to other countries. Our findings could be explained by the Chinese social insurance system and compulsory education policies. The compensation for treatment resources for children with epilepsy has led to a narrowing down of the medical resource gap and education gap between low-income and high-income populations. Low income is often associated with dropping out of school and interruption or stoppage of anti-epileptic drugs (AEDs), which in turn leads to a reduced QoL for the child with epilepsy (35). It has been reported that 50% of children with epilepsy in Uganda did not attend school and that children with epilepsy who drop out of school have a lower QoL than children who attend school (36). In contrast, although our cohort all had postoperative seizures, 92.7% of children were able to attend school normally, which brings increased opportunities for employment and higher QoL in the long term. Another probable explanation that can be offered is that these findings are a result of the selection bias, as all participants in the present study underwent resection surgery, and those who were extremely financially deprived were less likely to undergo surgery. Our patient selection may invisibly eliminate the impact of financial factors on QoL.

To be clear, the goal of epilepsy surgery is to improve the patient's QoL rather than just control seizures. Clinicians need to strike a proper balance between maximizing seizure control and improving the patient's quality of life by considering the potential for permanent neurological function deficits from resection surgery and the potential for drug overload to affect the child's intellectual development. The impact of epilepsy on QoL is closely related to seizure control, subjective feelings, and family (37), and the treatment for epilepsy requires not only seizure control but also the integration of clinicians, caregivers, and social support based on the anti-epileptic drug (AED) treatment to improve the patient's QoL.

### 4.1 Limitations

Some limitations should be acknowledged regarding our study. First, the present study is a single-center retrospective study with a relatively small sample size, which may result in false negative errors. Second, the cross-sectional design of this study did not allow us to obtain the variation in QoL with time. We chose annual household income and caregivers' education level as proxies for the effect of household disposable medical resources and parents' understanding of epilepsy on the QoL, but this may not be suitable in the long run. Despite its limitations, the present study is the first to explore the influencing factors of QoL in children with postoperative seizures and find that a lower ILAE seizure outcome classification is acceptable if seizure freedom is difficult to be achieved. This study also provides valuable ideas on how to improve the quality of life of children with postoperative seizures.

## 5 Conclusion

The measurement of QoL focuses on an individual's subjective perception of wellbeing and reflects patient's role functioning, overall health, and satisfaction with medical care. We found that the annual household income and surgery-related factors, such as surgical complications, surgical sequelae, and resection completion, made no significant difference in QoL for children with postoperative seizures. Presurgical language development delay or language dysfunction and the postsurgical ILAE seizure outcome classification can predict the QoL for children with epilepsy who have postoperative seizures. If seizure freedom is difficult to achieve after surgery, a lower ILAE seizure outcome classification, such as ILAE 1-3, is still an acceptable outcome, as it predicts a higher QoL.

## Data availability statement

The original contributions presented in the study are included in the article/Supplementary material, further inquiries can be directed to the corresponding author.

## Ethics statement

The studies involving human participants were reviewed and approved by Committee of Children's Hospital of Chongqing Medical University. Written informed consent to participate in this study was provided by the participants' legal guardian/next of kin.

## Author contributions

YW has made substantial contributions to the design of the manuscript. YW and ZZ acquired, analyzed, and interpreted the data. All authors participated in drafting the manuscript, and PL and XZ revised it critically, read, and approved the final version of the manuscript.
